# Symptom relief during last week of life in neurological diseases

**DOI:** 10.1002/brb3.1348

**Published:** 2019-07-09

**Authors:** Anneli Ozanne, Richard Sawatzky, Cecilia Håkanson, Anette Alvariza, Carl Johan Fürst, Kristofer Årestedt, Joakim Öhlén

**Affiliations:** ^1^ Institute of Health and Care Sciences, Sahlgrenska Academy University of Gothenburg Gothenburg Sweden; ^2^ Department of Neurology Sahlgrenska University Hospital Gothenburg Sweden; ^3^ School of Nursing Trinity Western University Langley British Columbia Canada; ^4^ Centre for Health Evaluation & Outcome Sciences St. Paul's Hospital Vancouver British Columbia Canada; ^5^ Department of Nursing Science Sophiahemmet University Stockholm Sweden; ^6^ Department of Health Care Sciences Palliative Research Centre, Ersta Sköndal Bräcke University College Stockholm Sweden; ^7^ Capio Palliative Care Dalen hospital Stockholm Sweden; ^8^ The Institute for Palliative Care Lund University and Region Skåne Lund Sweden; ^9^ Department of Clinical Sciences Lund University Lund Sweden; ^10^ Faculty of Health and Life Sciences Linnaeus University Kalmar Sweden; ^11^ The Research Section Kalmar County Council Kalmar Sweden; ^12^ Palliative Centre Sahlgrenska University Hospital Gothenburg Sweden; ^13^ The University of Gothenburg Centre for Person‐centred Care Gothenburg Sweden

**Keywords:** amyotrophic lateral sclerosis, brain neoplasms, end of life, motor neuron disease, neurological disease, palliative care

## Abstract

**Objectives:**

The aim of this study was to investigate symptom prevalence, symptom relief, and palliative care indicators during the last week of life, comparing them for patients with motor neuron disease (MND), central nervous system tumors (CNS tumor), and other neurological diseases (OND).

**Material & Methods:**

Data were obtained from the Swedish Register for Palliative Care, which documents care during the last week of life. Logistic regression was used to compare patients with MND (*n* = 419), CNS tumor (*n* = 799), and OND (*n* = 1,407) as the cause of death.

**Results:**

The most prevalent symptoms for all neurological disease groups were pain (52.7% to 72.2%) and rattles (58.1% to 65.6%). Compared to MND and OND, patients with CNS tumors were more likely to have totally relieved pain, shortness of breath, rattles, and anxiety. They were also more likely to have their pain assessed with a validated tool; to receive symptom treatment for anxiety, nausea, rattles, and pain; to have had family members receive end‐of‐life discussions; to have someone present at death; and to have had their family members offered bereavement support. Both patients with CNS tumor and MND were more likely than patients with OND to receive consultation with a pain unit and to have had end‐of‐life discussions.

**Conclusions:**

The study reveals high symptom burden and differences in palliative care between the groups during the last week of life. There is a need for person‐centered care planning based on a palliative approach, focused on improving symptom assessments, relief, and end‐of‐life conversations.

## 1 INTRODUCTION

Many neurological diseases—such as motor neuron disease (MND) (Ganzini, Johnston, & Silveira, 2002; Oliver et al., 2016), Parkinson's disease, other nervous system diseases (Tiirola, Korhonen, Surakka, & Lehto, 2017; Vorovenci, Biundo, & Antonini, 2016), and glioma (Koekkoek et al., 2014; Walbert & Chasteen, 2015)—are progressive and degenerative, leading to physical deterioration and increasingly distressing symptoms. Irrespective of diagnosis, symptoms such as pain, fatigue, dyspnea, rattles, anxiety, nausea, and confusion are prevalent at the end of life (Kehl & Kowalkowski, 2013; Öhlén et al., 2017). Other specific symptoms in neurological diseases include spasticity; paralysis; dysphagia and speech impairments; epileptic seizures; myoclonus; neuropsychological and neuropsychiatric impairments; recurring infections; aspiration pneumonia; and vegetative disorders (Golla et al., 2017; Oliver et al., 2016). Motor neuron disease (MND) and glioblastoma are well known for their aggressive illness trajectories; the average survival in MND is a few years (Wijesekera & Leigh, 2009) and the median survival in glioblastoma is just over one year (Stupp et al., 2005). Common symptoms in patients with MND include dyspnea, dysphagia, (Tiirola et al., 2017) fatigue (Tiirola et al., 2017; Xu et al., 2015), and weakness (Xu et al., 2015), while patients with glioblastoma often struggle with seizures, fatigue, depression, and anxiety (Walbert & Chasteen, 2015). One week before death, the most common symptoms in glioblastoma are somnolence, dysphasia, cognitive disturbances, motor deficit, headache, seizures (Koekkoek et al., 2014), and delirium (Pace et al., 2017).

Treatment of distressing symptoms in neurological diseases is an important focus throughout the illness trajectories and during end‐of‐life care. Two studies show that around 20%–35% of patients with MND undergo noninvasive ventilation (NIV) and around 35% receive enteral feeding at the end of life (Gil et al., 2008; Oliver et al., 2010). The latter study also reports that 66% use morphine, 77% midazolam, 53% anticholinergic medication, 37% glycopyrronium, and 19% hyoscine hydrobromide during the last 24 hr of life (Oliver et al., 2010). A study of 1603 older (≥65‐year‐old) patients with MND/amyotrophic lateral sclerosis (ALS) indicated that the five most commonly used drugs during the final month before death are psycholeptics, analgesics, laxatives, psychoanaleptics, and various other drugs affecting the nervous system. Medication for symptom control—including anxiolytics, sedatives, analgesics, antidepressants, gastroprotective agents, and anti‐emetics—is more prevalent during the last month of life (Grande, Morin, Vetrano, Fastbom, & Johnell, 2017).

Even though it is known that many neurological diseases experience increasing symptom burden as the illness progresses, little is known about symptom prevalence and the extent of symptom relief during the last week of life. To symptom relief in end‐of‐life care for people with neurological diseases, it is increasingly recommended that a palliative approach be centrally integrated into the care provided by all healthcare professionals' relief (Randall & Downie, 2006). Regardless of the diagnosis, a number of factors are important when integrating a palliative approach to care, including comprehensive assessment, patient and family engagement, communication, care coordination, attention to symptom relief, and preparation for an imminent death through end‐of‐life conversations with the dying person and family members (Ferrell, Twaddle, Melnick, & Meier, 2018). It is therefore important to study symptom relief in palliative care at the end of life (De Roo et al., 2013) and also to obtain such knowledge from a population perspective (Cohen & Deliens, 2012). Although disease progression is often more aggressive for MND and CNS tumors than for other neurological diseases, there is limited knowledge about symptom prevalence and relief among these disease groups at end of life. In addition, since palliative care knowledge is historically primarily focused on care of patients with cancer, there is a gap in knowledge about differences in a palliative approaches to care across the different disease groups. Better knowledge about possible differences might give rise to strategies to improve the care regardless of the disease in question.

Thus, the aim of this study was to investigate symptom prevalence, symptom relief, and palliative care during the last week of life, by comparing the experiences of patients with neurological diseases grouped as MND, CNS tumor, and other neurological diseases (OND).

## 2 MATERIAL & METHODS

### 2.1 Study population and data collection

This was a retrospective register study. Data were obtained between 1 January 2011 and 31 December 2012 from the Swedish Register for Palliative Care (SRCP) (http://palliativ.se), which is a national quality register documenting care during the last week of life, irrespective of diagnosis (Lundström, Axelsson, Heedman, Fransson, & Fürst, 2012). During this period, the register covered 53% and 62% of all deaths in Sweden for 2011 and 2012, respectively (Öhlén et al., 2017). Information for the register was obtained from healthcare professionals (physician, nurse, or team) based on their responses to a questionnaire completed after the patient's death. Inclusion criteria were adult patients (≥18 years old) who had neurological diseases or CNS tumors as the cause of death. The Swedish Causes of Death Certificate Register (covering all deaths) was used to identify patients confirmed to have MND, CNS tumor, and OND, as classified according to the English version of the International Statistical Classification of Diseases and Related Health Problems (ICD‐10), and reported by physicians. The following ICD‐10 codes were used to identify patients: G122 for MND; C700–C729 for CNS tumor, and G00–G99 for OND. Exclusion criteria from the group of OND G00–G99 were G30–G32 (other degenerative diseases in the nervous system, e.g., Alzheimer's disease). C700–C729 includes malignant tumors in the CNS membrane of the brain, in the brain itself, and in the spinal cord, cranial nerves, or other parts of the CNS.

For the purpose of estimating representativeness of the sample, population‐level data were retrieved from the Swedish Causes of Death Certificate Register pertaining to cause of death based on death certificates (reported by physicians) for all individuals with MND, CNS tumor, and OND. Data included information about adults (≥18 years old) during 2011–2012 (The Swedish National Board of Health & Welfare, 2018).

### 2.2 Study variables

Selected variables from the SRCP were organized into patient characteristics, symptom presence and symptom relief, and key indicators of palliative care. Variables for patient characteristics were age, sex, place of death, and type of care setting, as well as the number of days enrolled in the service. Place of death and type of care setting were categorized into general home care, short**‐**term care facility for elderly people, hospital ward/department, specialized palliative home care, hospice or palliative care unit, long‐term care for older people, and other. Presence and degree of relief were assessed for the following symptoms: pain, nausea, shortness of breath, rattles, anxiety, and confusion. Each symptom was reported as “not present,” “totally relieved,” “partly relieved,” “not relieved at all,” or “unknown.” Assessments of pain were based on use of validated tools in clinical practice, such as visual analog scale, numeric rating scale, and Abbey pain scale. Information about individual prescription of pro re nata injections (PRN injections) was documented for the each of the above symptoms, except for shortness of breath and confusion. Also included were time since last examination by a physician, consultation of external expertise for symptom relief (e.g., pain or palliative care team), presence of someone at the time of death, patient's death occurring in preferred place, and whether bereavement support for family members had been offered. Variables pertaining to end‐of‐life conversations with patients and family members were not limited to the last week of life. These variables were entered as “yes,” “no,” or “unknown.”

### 2.3 Statistical analysis

Descriptive statistics were used to present patient characteristics, symptom presence, symptom relief, and palliative care during the last week of life from the SCRP, as well as data from the Swedish Causes of Death Certificate Register.

Following well‐established procedures for categorical data analysis (Agresti, 2010), logistic regression models were used to compare symptoms, symptom relief, and palliative care indicators among the three patient groups, while adjusting for covariates (age, sex, place of death, days enrolled in the service). Separate multinomial logistic regression analyses were completed for each symptom as a dependent variable with categories “no,” “yes, but totally relieved,” and “yes, partly relieved or not relieved at all” (the last two categories were collapsed due to sparse data). Similarly, multinomial logistic regression analyses were completed for dummy‐coded palliative care indicators that had more than two categories, and logistic regression models were used for indicators that had only two categories. Comparisons were made by including neurological diseases (MND, CNS tumor, OND) as dummy‐coded independent variables, together with the covariates. The adjusted odds ratios (ORs) are reported as forest plots to facilitate interpretation and comparison across the three diagnostic groups.

Multiple imputation was used to accommodate missing data, and results were compared to these based on complete case analysis (Little & Rubin, 2019). We imputed 15 data files using expectation–maximum likelihood imputation with all variables included as covariates, as implemented in SPSS. Statistical significance was defined as *p* < 0.05. The statistical analyses were performed using SPSS Statistics version 23 (IBM Corp).

The Regional Ethical Review Board in Stockholm granted ethical approval for the study (approval: 2013/1576‐31/3).

## 3 RESULTS

### 3.1 Background characteristics

Of the 105,229 registered patients in the SRCP in 2011 and 2012, 2,627 patients ≥18 years old were registered with MND (*n* = 419/15.9%), CNS tumor (*n* = 799/30.4%), or OND (*n* = 1409/53.6%) as underlying cause of death. The SRCP does not cover all patients in Sweden and a report from the Swedish national board of health and welfare recorded prevalence of the underlying cause of death for adults as 7.7% for MND, 12.0% for CNS tumor, and 80.3% for OND in 2011–2012 (The Swedish National Board of Health & Welfare, 2018).

Data from the SRCP showed that 29.8% of the patients with MND and 26.8% with OND died in a hospital ward/department, while this figure was only 14.6% in patients with CNS tumor. In comparison, 31.0% of patients with CNS tumor died in a hospice or palliative care unit, while this figure was 15.0% in the case of MND and 2.3% for OND. Further background characteristics are presented in Table 1.

**Table 1:** Background characteristics of the 2,627 participants

Variables	MND	CNS tumor	OND
Sex: male/female *n* (%)	199/220(47.5/52.5)	466/333(58.3/41.7)	719/690(51.0/49.0)
Age: mean/*SD*	70.6/10.9	64.8/14.1	77.2/12.6
Number of days enrolled to the service: md	32	29	342
Place of death			
Home; general home care	46 (11.0)	50 (6.3)	85 (6.0)
Short‐term care facility for elderly people	30 (7.2)	140 (17.5)	73 (5.2)
Hospital ward/department	125 (29.8)	117 (14.6)	378 (26.8)
Specialized palliative home care	80 (19.1)	167 (20.9)	43 (3.1)
Hospice or palliative care unit (inpatient)	63 (15.0)	248 (31.0)	33 (2.3)
Long‐term care for elderly people	70 (16.7)	74 (9.3)	782 (55.5)
Other	5 (1.2)	3 (0.4)	15 (1.1)
Total group	419	799	1,409

Abbreviations: CNS tumor, central nervous system tumors; MND, motor neuron disease; OND, other neurological diseases.

### 3.2 Symptoms, symptom relief, and palliative care indicators

**Figure 1:**
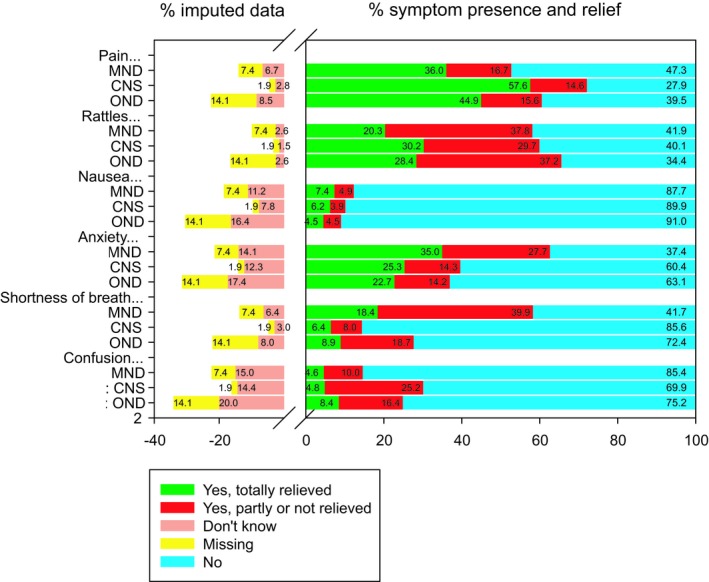
Symptom prevalence presence and relief. Note: Positive values indicate estimated prevalence percentages out of the total sample after multiple imputation (*n* = 2,627). Negative values indicate percentages of imputed data. CNS, central nervous system tumours; MND, motor neuron disease; OND, other neurological diseases

Pain (52.7%–72.2%) and rattles (58.1%–65.6%) were the most prevalent symptoms, and nausea (9.0%–12.3%) was the least prevalent symptom in all three study groups (Figure 1). Anxiety (62.7%) and shortness of breath (58.3%) were also among the most prevalent symptoms in patients with MND, as was confusion (30.0%) in patients with CNS tumor. None of these were reported as being totally relieved. While pain (36%–57.6%) was the symptom with the highest percentage of being totally relieved (in all three groups), shortness of breath (39.9%) had the highest percentage of not being totally relieved in MND. Rattles (29.7%–37.8%) was also a symptom with a relatively high percentage of not being totally relieved in all three study groups, as was confusion in CNS tumor (25.2%).

Palliative care indicators are described in Table 2. In general, patients with CNS tumor were more likely to receive PRN prescriptions than patients with MND or OND.

**Table 2:** Palliative care in patients with MND, CNS tumor, and OND

Variables	MND (*n* = 419)	CNS tumor (*n* = 799)	OND (*n* = 1,407)
End‐of‐life discussions			
With patient			
Yes %	68.8	72.8	32.1
No %	31.2	27.2	67.9
Missing (%)	(19.8)	(16.5)	(27.2)
With family member(s)			
Yes %	77.4	89.3	68.7
No %	16.5	7.4	24.2
Had no relatives %	6.1	3.3	7.1
Missing (%)	(17.7)	(9.5)	(20.8)
Time since last examination of a physician			
Day/days %	75.2	75.3	63.1
Week/weeks %	18.3	19.9	23.8
Month/months %	6.5	4.8	13.1
Missing (%)	(9.1)	(3.3)	(17.0)
External expertise consultation			
Pain unit			
Yes %	1.2	0.8	0.4
No %	98.8	99.2	99.6
Missing (%)	(0.0)	(0.0)	(0.0)
Palliative care team			
Yes %	16.9	15.1	2.3
No %	83.1	84.9	97.7
Missing (%)	(0.0)	(0.0)	(0.0)
Other hospital units			
Yes %	16.5	6.8	7.2
No %	83.5	93.2	92.8
Missing (%)	(0.0)	(0.0)	(0.0)
Pain assessment performed			
Yes %	15.4	22.5	12.6
No %	84.6	77.5	87.4
Missing (%)	(14.6)	(6.3)	(20.6)
PRN prescriptions			
For pain			
Yes %	85.2	95.3	86.2
No %	14.8	4.7	13.8
Missing (%)	(8.4)	(2.1)	(15.7)
For rattles			
Yes %	83.7	90.6	83.0
No %	16.3	9.4	17.0
Missing (%)	(8.4)	(2.4)	(15.9)
For nausea			
Yes %	61.5	75.8	42.7
No %	38.5	24.2	57.3
Missing (%)	(9.8)	(3.0)	(17.2)
For anxiety			
Yes %	84.3	90.2	73.2
No %	15.7	9.8	26.8
Missing (%)	(9.1)	(2.6)	(16.5)
Intravenous fluid or nutritional support last day of life			
Yes %	39.6	7.1	20.6
No %	60.4	92.9	79.4
Missing (%)	(9.1)	(2.0)	(15.5)
Wishes met regarding desired place of death			
Yes %	81.7	83.7	81.5
No %	18.3	16.3	18.5
Missing (%)	(36.3)	(37.2)	(62.5)
Someone present at death			
Nobody %	14.8	8.2	17.8
Staff %	20.1	16.5	32.5
Relatives %	38.7	48.5	27.8
Staff and relatives %	26.4	26.9	22.0
Missing (%)	(1.4)	(0.4)	(2.9)
Bereavement support offered to family member(s)			
Yes %	73.3	83.5	58.2
No %	26.7	16.5	41.8
Missing (%)	(22.4)	(13.0)	(27.5)

Note

Percentages for valid responses are multiple imputation estimates based on the total sample. Missing data percentages are reported in brackets.

Abbreviations: CNS tumor, central nervous system tumors; MND, motor neuron disease; OND, other neurological diseases.

### 3.3 Comparison between patients with MND, CNS tumor, and OND

**Figure 2:**
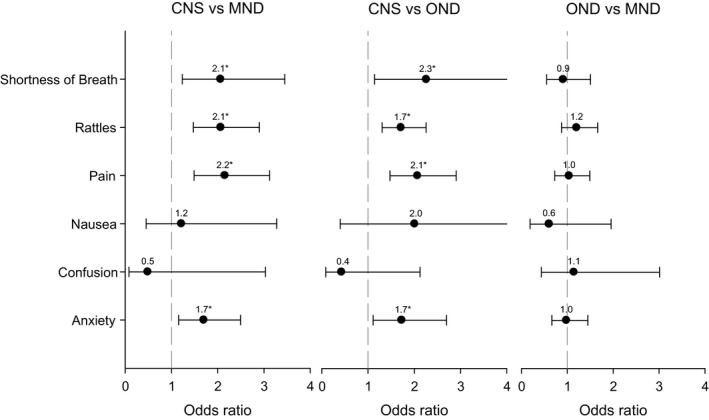
Odds ratios for symptom relief. Note: Results based on multinomial logistic regression and multiple imputation (*n* = 2,627). Odds ratios compare “yes, but totally relieved” to “yes, partly or not relieved” and are adjusted for age, sex, place of death, days enrolled in the service. Error bars indicate 95% confidence interval. *p < 0.05. CNS, central nervous system tumours; MND, motor neuron disease; OND, other neurological diseases

Compared to MND and OND, patients with CNS tumor were more likely to have their pain, shortness of breath, rattles, and anxiety totally relieved, rather than partly or not at all relieved, with odds ratios ranging from 2.3 to 1.7 (Figure 2). There were no statistically significant differences in the odds ratios comparing patients with MND and OND.

**Figure 3:**
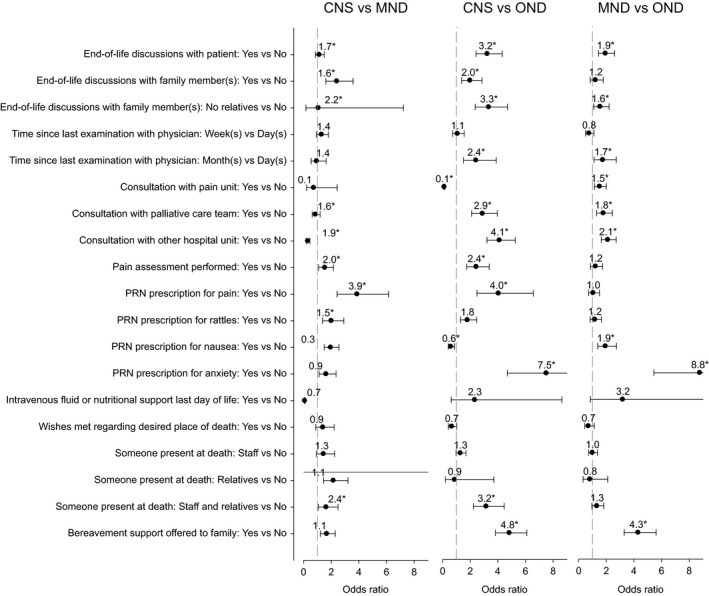
Odds ratios for palliative care indicators. Note: Results based on multinomial logistic regression and multiple imputation (*n* = 2,627). Odds ratios are adjusted for age, sex, place of death, days enrolled in the service. See Table 2 for % missing on each of the variables. Error bars indicate 95% confidence interval. *p < 0.05. CNS tumour, central nervous system tumours; MND, motor neuron disease; OND, other neurological diseases

With regard to the palliative care indicators, compared to patients with MND and OND, patients with CNS tumor were more likely to have their pain assessed with a validated tool; to receive symptom treatment for anxiety, nausea, rattles, and pain; to have family members receive end‐of‐life discussions; to have someone present at death; and to have bereavement support offered to family members. The most substantial difference for patients with CNS tumor was for prescribed pain medications, with odds ratios of 3.9 (MND) and 4.0 (OND). Both patients with CNS tumor and MND were more likely than patients with OND to receive consultation with a palliative care team (OR = 7.5 and 8.8, respectively), to receive end‐of‐life discussions from health professionals (OR = 4.8 and 4.3, respectively), and to have their wishes met regarding desired place of death (OR = 2.4 and 1.7, respectively). Conversely, patients with OND were substantially more likely than patients with CNS tumor to receive intravenous fluid or nutritional support on the last day of life (OR = 8.6). In addition, compared to OND, patients with MND were more likely to have PRN prescriptions for nausea (OR = 2.1) and anxiety (OR = 1.8); to have family members present at death (OR = 1.6); and to have had family members offered bereavement support after the patient's death (OR = 1.9; Figure 3).

## 4 DISCUSSION

The prevalence of severe symptoms and lack of symptom relief that was found in the present study suggests that patients with neurological diseases might not receive adequate palliative care during their last week of life. Regardless of diagnosis, our study showed that the patients had symptoms such as shortness of breath, rattles, and pain. The number of patients who were not relieved or were only partly relieved from the severe symptoms varied. Comparison among the three studied groups revealed that the patients with OND received less palliative care compared to the patients with MND and CNS tumor, for example, regarding prescription for anxiety and nausea; consultation with pain unit; and end‐of‐life discussions. Prescriptions to treat anxiety, pain, rattles, and nausea were also more prevalent for patients with CNS tumor than for MND. The high levels of symptom distress, as well as the differences between the three groups studied, require attention to ensure that equal care can be given regardless of the diagnosis.

In accordance with earlier studies from the SRCP, examining other diagnoses and specific types of care, the prevalence of symptoms was high during the last week of life (Andersson, Årestedt, Lindqvist, Fürst, & Brännström, 2018; Årestedt et al., 2018; Axelsson et al., 2018; Smedbäck et al., 2017). It is not surprising that, for example, shortness of breath and rattles were common symptoms in MND, since the major direct causes of death are pneumonia and respiratory failure (Cheng et al., 2018; Gil et al., 2008). However, it is problematic that—in the last week of life—the percentage of patients who were not relieved from symptoms such as shortness of breath, rattles, anxiety, and pain was so high, irrespective of having MND, CNS tumor, or OND. One explanation might be that the number of patients who received care from specialist palliative care teams or received consultation with external competences was very low in all the groups studied. Since it is known that multidisciplinary palliative care teams can manage symptoms and other issues effectively (Oliver et al., 2016), it would be useful to have more active involvement and cooperation of consulting specialist palliative care teams in the care of these patients.

Depending on the diagnoses, the progression of symptoms and its rapidity differ between the three studied groups. Health service professionals' knowledge about OND and its trajectory might be insufficient (especially since 55.5% of these patients lived at long‐term care facilities for older people) and the symptoms might not be noted as in more aggressive diseases like MND or CNS tumor. It might also suggest that the patients with OND did not receive the same degree of palliative care as the other groups studied. Another possible explanation for the differences might be that many neurological diseases have a chronic course with longer disease trajectories than for MND (Wijesekera & Leigh, 2009) and many cancer diagnoses (Golla et al., 2017). The differences in the number of days enrolled to the service between the groups of MND and CNS tumor versus OND probably depend on differences in disease trajectories and progression in MND and CNS as compared to OND. Consequently, it might be difficult to assess symptoms and decide when patients are in need of palliative care and when to give appropriate treatment. As recommended based on previous research (Oliver et al., 2016), a focus on a palliative approach to care, also at an earlier stage, might lead to higher attention being paid to what type of care is appropriate. Person‐centeredness combined with a palliative approach to care is necessary to focus on individual differences in symptoms and symptom relief in striving for equal care.

Only around 32% of the patients with OND were offered end‐of‐life discussions, while the figure was around 69% in the case of their family members. It may be that these numbers are related to cognitive or consciousness decline and similar results are found for other diagnoses (Årestedt et al., 2018; Axelsson et al., 2018). It would be interesting to explore why patients do not receive this discussion. It is important to offer participation in end‐of‐life discussions as such interaction represents an essential part of palliative care (Ferrell et al., 2018). Having end‐of‐life discussions with patients and family members is a significant prerequisite for professionals to learn more about the patient's personal preferences regarding both place and planning of care at the end of life.

To our knowledge, there is a lack of studies focusing on patients with neurological diseases grouped into MND, CNS tumor, and OND, examining and comparing symptom prevalence and relief, as well as palliative care during the last week of life. The goal of the SRCP is to improve the quality of care at the end of life (Lundström et al., 2012), and this register study provides knowledge that could be used for such improvement. This is the first study on neurological diseases based on SRCP data, focusing specifically on diseases from the central and peripheral nervous system, as well as degenerative and nondegenerative diseases. In future studies, one should consider to define and separate the disease groups further. It should be taken into account that data from the SRCP do not cover all deaths and, therefore, the results do not represent all patients. For the future development of this type of quality registry, it would be beneficial to also include patients' prospectively reported experience and outcome data. In addition, as described in the background, patients with MND and CNS tumor also have other symptoms, such as dysphagia, dysarthria, and spasticity, which are not measured by the register. It is plausible that, even though they are not included in the register themselves, these distressing symptoms may affect the symptoms that *are* included.

The questionnaire for the register was completed retrospectively by a responsible or special assigned clinician (physician or registered nurse). Of course, answers might differ dependent on how engaged the clinician had been in individual patient's care and how long after the patient's death the questionnaire was completed. In addition, the % missing data were quite large for several of the variables. Multiple imputation was used to obtain the most defensible estimates accommodating missing data. However, these estimates should nonetheless be interpreted with caution for variables that have greater percentages of missing data. The resulting uncertainty is reflected in wider confidence intervals for these variables (as shown in Figures 2 and 3). It is recommended to improve the reporting on of these variables in the SRCP to as to enhance the ability to obtain more.

In conclusion, the study indicates high symptom burden in the last week of life for patients dying from neurological diseases. These patients do not receive the palliative care that they need. Person‐centered communication and care planning, symptom assessment, and end‐of‐life conversations with both patients and family members, including a special emphasis on active symptom relief practices, are clearly needed.

## CONFLICTS OF INTERESTS

None of the authors have any conflicts of interest to disclose.

## DATA AVAILABILITY STATEMENT

The data that support the findings of this study are available on request from the corresponding author.
